# The global burden of aortic aneurysm attributable to smoking from 1990 to 2021: Current trends and projections for 2036

**DOI:** 10.18332/tid/215179

**Published:** 2026-02-17

**Authors:** Xiaomin Jin, Dingwen Xu, Xiang Lian, Xueyu Geng, Zejun Yue, Weijuan Yao, Xifu Wang

**Affiliations:** 1 Department of Emergency, Beijing Anzhen Hospital, Capital Medical University, Beijing, China; 2 Tongzhou District Hospital of Integrated Traditional Chinese and Western Medicine, Beijing, China; 3 Department of Clinic, School of Medicine, Yangzhou Polytechnic College, Yangzhou, China; 4 Department of Physiology and Pathophysiology, School of Basic Medical Sciences, Peking University Health Science Center, Beijing, China

**Keywords:** aortic aneurysm, smoking, global burden of disease, estimated annual percentage change (EAPC), autoregressive integrated sliding average (ARIMA) model

## Abstract

**INTRODUCTION:**

Smoking is a major risk factor for aortic aneurysm (AA). This cross-sectional study used Global Burden of Disease (GBD) data to examine trends in smoking-related AA and project future trends.

**METHODS:**

We conducted a secondary analysis on the GBD 2021 dataset. Data on mortality, disability-adjusted life years (DALYs), age-standardized mortality rate (ASMR), and age-standardized DALYs rate (ASDR) associated with AA attributable to smoking from GBD. The estimated annual percentage change (EAPC) was employed to assess burden trends from 1990 to 2021. The autoregressive integrated sliding average (ARIMA) model was utilized to project disease trends from 2022 to 2036. Decomposition analysis and frontier analysis were employed to comprehensively examine the data.

**RESULTS:**

Globally in 2021, there were 47538 deaths (95% UI: 39480–56008) from smoking-attributable AA, with an ASMR of 0.56 per 100000 (95% UI: 0.46–0.66) and 1157488 DALYs (95% UI: 989966–1325831). The ASMR showed a decreasing trend from 1990 to 2021 (EAPC= -2.17; 95% CI: -2.30 – -2.04). However, there were significant differences between regions, with the burden of deaths in high sociodemographic index (SDI) areas accounting for 40.3% of the global total, while ASMR in low-middle SDI areas rose (EAPC=0.99; 95% CI: 0.94–1.03). The point estimates for ASMR and ASDR were consistently greater in males compared to females throughout the study period. Projections indicate a continued global decline in ASMR and ASDR over the next 15 years, but the burden would gradually rise in Central Asia, East Asia, and South Asia.

**CONCLUSIONS:**

Smoking remains a significant preventable risk factor for AA, with substantial sex and regional disparities. Targeted public health strategies are urgently needed to address rising burdens in specific regions and reduce health inequalities.

## INTRODUCTION

Aortic aneurysm (AA) is a serious vascular disease that leads to local dilatation of the aorta, which is characterized by local dilatation of the aorta and can lead to fatal bleeding, causing significant harm and economic burden to patients and society^1^. Several studies have identified smoking history, hypertension, and male gender as major risk factors contributing to the burden of this disease^2-4^. Among many risk factors, smoking is established as the strongest risk factor, and studies have shown that smokers have a 5.23-fold increased risk of AA compared with non-smokers, which is significantly higher than hypertension^5^. Although the current treatment of aortic aneurysms mainly includes surgical and medical treatment, there are still significant limitations in the early detection rate and treatment outcome^6^. Therefore, it is particularly important to deeply investigate the impact of smoking on the burden of aortic aneurysm disease and provide a basis for formulating effective public health policies.

Smoking has been shown to be a major risk factor for a number of cardiovascular diseases, including aortic aneurysms^7^. Mechanistic studies suggest that smoking is associated with vascular endothelial injury and the progression of arteriosclerosis, biological pathways linked to an increased risk of aortic aneurysm development^8,9^. Despite this, current systematic analyses of the impact of smoking on aortic aneurysm burden remain inadequate. Existing literature is mostly focused on a single region or specific population and there is a lack of comparative studies worldwide. Therefore, this study aims to fill this gap and assess the impact of smoking on the burden of aortic aneurysms worldwide and across regions.

This study aimed to quantify global and regional trends in aortic aneurysm burden attributable to smoking from 1990 to 2021 and to project future patterns up to 2036 using the GBD 2021 data. The findings may offer valuable insights for informing public health strategies aimed at reducing smoking-related AA burden.

## METHODS

### Data sources

In this study, the Global Burden of Disease (GBD) 2021 dataset^10^ was used for a secondary analysis. GBD 2021 dataset includes a comprehensive assessment of 369 diseases, injuries, and health conditions and 88 risk factors in 204 countries and regions. GBD assessment data are generated through a standardized analysis system that integrates multiple data sources, including vital registration systems, health surveys, and hospital records. This study provides insight into the burden of aortic aneurysm disease associated with smoking based on up-to-date epidemiological data and well-established standardized methods. It is available through the Global Health Data Exchange (GHDx) online platform, which provides critical insight into understanding global health trends. With this platform, we selected disease categories, associated risk factors, and demographic variables that were compatible with the study objectives. The methodological framework used for data collection and processing has been elaborated in previous literature. We extracted data on annual deaths, disability-adjusted life years (DALYs), and age-standardized rates (ASRs) associated with aortic aneurysms between 1990 and 2021 from the GBD 2021 dataset^11,12^. The GBD study employs the Comparative Risk Assessment (CRA) framework to estimate the burden of disease attributable to risk factors. For smoking, this involves calculating the PAF based on the relative risks of AA for smokers versus non-smokers, derived from meta-analyses of epidemiological studies, and the prevalence of smoking exposure. The number of deaths and DALYs attributable to smoking were then calculated by multiplying the PAF by the total AA burden. The age-standardized mortality rate (ASMR) and age-standardized DALYs rate (ASDR) attributable to smoking were computed by applying the PAF to the overall ASMR and ASDR, respectively, using the GBD world standard population.

### Sociodemographic index (SDI)

Sociodemographic index (SDI), as a comprehensive development indicator, is closely related to health outcomes. The index consists of geometric means of three key components, each standardized on a scale of 0 to 1: female fertility below 25 years of age, average years of education of the population aged ≥15 years, and lagged adjusted per capital income. In the GBD 2021 analysis, SDI scores are shown on an adjusted scale (i.e. raw index multiplied by 100). Thus, a score of 0 represents the lowest level of development, while a score of 100 represents the highest level of development. According to their SDI values, the 204 countries and regions covered in the study were divided into five groups: low, low-middle, middle, high-middle, and high. This classification helps to understand the relationship between development and health in different regions^13^.

### ARIMA model prediction

The Autoregressive Integrated Moving Average (ARIMA) model, a widely used method for time series analysis, was employed for forecasting. This model combines autoregressive (AR), differencing (I), and moving average (MA) components. We applied the ARIMA model to the original level of the age-standardized rates. The time series was first differenced to achieve stationarity, which was confirmed using the Kwiatkowski-Phillips-Schmidt-Shin (KPSS) test. The optimal model orders (p, d, q) were selected by comparing models based on the minimization of the Akaike information criterion (AIC) and Bayesian information criterion (BIC). The Ljung-Box test was used to confirm the residuals exhibited no significant autocorrelation, indicating a good model fit. To internally validate the model’s predictive performance, we conducted a back-testing procedure, the model was fitted on data from 1990 to 2011 and used to forecast the period 2012–2021. When the residuals of the ARIMA model presented randomness (white noise) characteristics, the model was considered as the best linear predictor for short-term time series prediction^14^.

### Cross-national health inequality analysis

This study used the World Health Organization (WHO) defined inequality slope index (SII) and concentration index to assess the absolute and relative degree of inequality in the burden of aortic aneurysm disease caused by smoking. SII was derived by correlating disability-adjusted life years (DALYs) rates with sociodemographic indices (SDI) based on the median cumulative population distribution stratified by SDI and using a weighted regression model to correct for heteroscedasticity; concentration indices were determined by calculating the area under the Lorentz concentration curve by numerical integration, which was constructed based on the relative cumulative distribution of populations ranked by SDI and their corresponding cumulative distribution of DALYs. SII represents the health gap between countries with the highest SDI and the lowest SDI, with negative SII indicating that groups with higher SDI have lower disease burden; when Lorentz curves lie above the line of equality, the concentration index is negative, indicating that disease burden is concentrated in areas with lower SDI and vice versa^15^. Population weights were applied in the calculation of the SII and concentration index to ensure estimates represented population-weighted disparities rather than simple inter-country averages. The 95% UIs for the concentration index was propagated from the 1000 draws of the GBD 2021 estimation process, capturing the uncertainty inherent in the underlying ASMR and ASDR data.

### Frontier analysis

This study used frontier analysis methods in order to assess the relationship between health performance and social development level in various countries. A longitudinal dataset of SDI versus ASDR was constructed based on global burden of disease data from 1990–2021. Optimal efficiency fronts were identified by performing 100 repetitions of non-parametric bootstrap sampling, i.e. for each SDI level, the minimum value of ASDR was calculated as the frontier value at that level. The gap between the observed ASDR and their corresponding cutting-edge values at the SDI level was then calculated across countries (efficiency gap, effdiff = ASDR - frontierASDR). To reveal the heterogeneity characteristics in the process of health transition, the analysis focused on three typical types of cases: 1) 15 countries with the largest efficiency gap; 2) low SDI countries with low SDI (SDI <0.5) but excellent efficiency performance; and 3) high SDI countries with high SDI (SDI >0.85) but abnormal efficiency performance^16^.

### Statistical analysis

The main indicators we assessed for the burden of aortic aneurysm disease included: the number of deaths, mortality, disability-adjusted life years (DALYs), age-standardized mortality rate (ASMR), and age-standardized disability-adjusted life rate (ASDR). All rates were reported per 100000 population. For decomposition analysis, we applied the Das Gupta method to decompose the changes in age-standardized mortality rate (ASMR) and age-standardized DALYs rate (ASDR) into contributions from population growth, population aging, and epidemiological trends. This method allows for a standardized quantification of each factor’s impact, aligning with previous Global Burden of Disease studies. The uncertainty intervals (UIs) for all estimates, including deaths, DALYs, ASMR, and ASDR, were derived using the GBD’s standard methodology. This process involves accounting for uncertainty from multiple sources, such as sampling error, model specification, and data adjustment factors. The final uncertainty interval (typically the 25th and 975th values of 1000 draws) represents the 95% UI around the mean estimate. The estimated annual percentage change (EAPC) was calculated by fitting a linear regression to the natural logarithm of the annual ASRs, and its 95% confidence interval (CI) was obtained from the standard error of the regression coefficient. Age-standardized rate (ASR) was calculated as the ratio per 100000 population using the following formula: (age-specific rate in the first age group; number of people in the corresponding age group in the standard population; A: number of age groups). Estimating annual percent change (EAPC) is a key indicator in epidemiology to assess time trends in age-standardized rates of disease (ASRs) and is estimated using a linear regression framework in which regression coefficients (symbolized as β) are derived from natural logarithms of ASRs. In this model, y represents ln (ASR), while x represents calendar year. The 95% CI of the regression coefficient β can be calculated from its estimate and standard error (SE); the 95% CI of the EAPC can be obtained by substituting the upper and lower 95% CI of β into the EAPC calculation formula, respectively. The EAPC is presented with its 95% CI to provide a comprehensive perspective on time trends: Y = α + βx + ε; EAPC =100×[exp (β) - 1]. A lower bound of the 95% CI >0 indicates an upward trend; an upper bound <0 indicates a downward trend; and conversely, a value of 0 falling within the 95% CI indicates no statistically significant change in the trend. All statistical analyses were done using R software^16^ (version 4.4.1) combined with Joinpoint software^17^ (version 4.9.1.0, National Cancer Institute Monitoring Research Program Development). The threshold for statistical significance was set at p<0.05.

## RESULTS

### Global, regional, and national burdens and time trends of smoking-related aortic aneurysms

**Figure 1:**
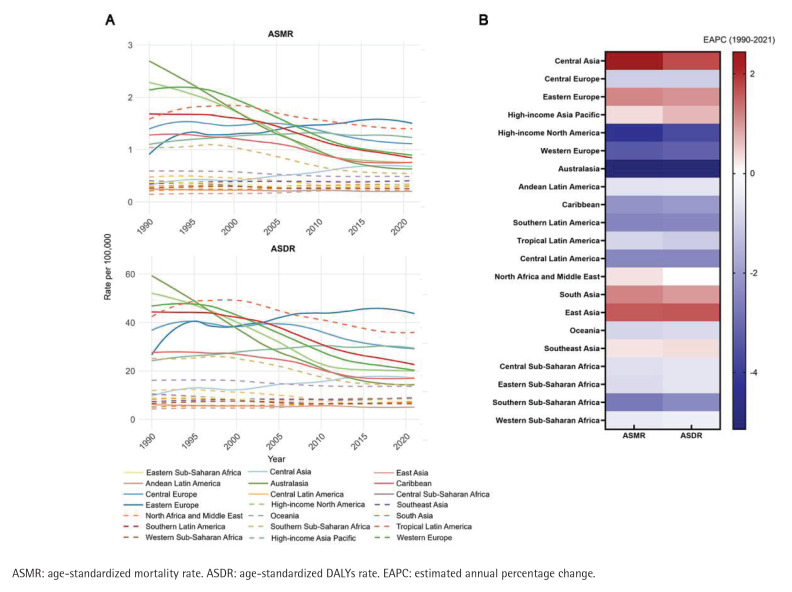
Trends in ASMR and ASDR of aortic aneurysm due to smoking and corresponding EAPC levels in different parts of the world from 1990 to 2021: A) ASMR and ASDR trends; B) EAPC levels

In 2021, the number of smoking-related aortic aneurysm deaths worldwide was 47538 (95% UI: 39480–56008), the ASMR was 0.56/100000 (95% UI: 0.46–0.66) (Table 1); the DALYs of smoking-related aortic aneurysms were 1157488 (95% UI: 989966–1325831), and the ASDR was 13.35/100000 (95% UI: 11.40–15.31) (Table 2). Between 1990 and 2021, the burden of smoking-related aortic aneurysms showed a decreasing trend (EAPC for ASMR: -2.17; 95% CI: -2.30 – -2.04; EAPC for ASDR: -1.90, 95% CI: -2.01 – -1.78) (Tables 1 and 2). At the regional level in 2021, the highest point estimate for ASMR was observed in Eastern Europe (1.50/100000; 95% UI: 1.28–1.72), followed by tropical Latin America and high-income countries in the Asia-Pacific region; while the lowest point estimate for ASMR was seen in Andean Latin America (0.20/100000; 95% UI: 0.15–0.27). Between 1990 and 2021, there were significant differences in the annual rate of change (EAPC) of ASMR between GBD regions: ASMR and ASDR showed an increasing trend in Central Asia, East Asia, South Asia, Eastern Europe, and high-income Asia-Pacific regions, and a steady or decreasing trend in the rest of the regions. Central Asia had the highest EAPC value (2.44; 95% CI: 2.22–2.67), followed by East Asia (1.58; 95% CI: 1.39–1.77); the region with the greatest magnitude of change was Australia, whose EAPC for ASMR was -5.14 (95% CI: -5.32 – -4.97) (Figure 1 and Table 1). The highest EAPC value for ASDR occurred in Central Asia (1.73; 95% CI: 1.52–1.95) and the lowest value in Australia (-5.04; 95% CI: -5.22 – -4.86) (Figure 1 and Table 2). Based on SDI regional analysis, the high SDI region accounted for 40.3% (19148 cases, 95% UI: 15517–23321) of aortic aneurysm-related deaths worldwide in 2021, and its ASMR was the highest (0.91; 95% UI: 0.75–1.09); notably, disease burden varied significantly between SDI regions. In contrast to the decreasing trend in the high-middle SDI regions, the age-standardized rates increased in the middle, low-middle and low SDI regions, with the greatest magnitude of increase in EAPC was observed in the low-middle SDI regions (EAPC for ASMR: 0.99; 95% CI: 0.94–1.03; EAPC for ASDR: 0.91; 95% CI: 0.86–0.95) (Supplementary file Figure S1).

**Table 1:** Deaths and ASMR of aortic aneurysm due to smoking in 1990 and 2021, and EAPC from 1990 to 2021

*Location*	*Deaths 1990* *Number* *(95% UI)*	*ASMR 1990* *Rate/100000* *(95% UI)*	*Deaths 2021* *Number* *(95% UI)*	*ASMR 2021* *Rate/100000* *(95 % UI)*	*EAPC* *(95 % CI)*
**Global**	36485 (31090–42554)	0.98 (0.82–1.15)	47538 (39480–56008)	0.56 (0.46–0.66)	-2.17 (-2.30 – -2.04)
**SDI Regions**					
High	23031 (19620–26755)	2.04 (1.74–2.36)	19148 (15517–23321)	0.91 (0.75–1.09)	-3.04 (-3.20 – -2.88)
High-middle	7858 (6877–8871)	0.80 (0.69–0.90)	13062 (11071–14998)	0.67 (0.56–0.76)	-0.92 (-1.11 – -0.74)
Middle	3538 (2921–4320)	0.36 (0.30–0.45)	9138 (7605–10699)	0.35 (0.29–0.41)	-0.47 (-0.59 – -0.35)
Low-middle	1468 (980–2442)	0.26 (0.17–0.43)	4826 (3682–7248)	0.35 (0.26–0.53)	0.99 (0.94–1.03)
Low	533 (236–936)	0.25 (0.11–0.44)	1301 (698–2150)	0.27 (0.15–0.45)	0.14 (-0.08–0.36)
**Central Asia, Central and Eastern Europe**					
Central Asia	161 (137–197)	0.33 (0.28–0.40)	542 (448–638)	0.67 (0.56–0.79)	2.44 (2.22–2.67)
Central Europe	2054 (1807–2311)	1.38 (1.21–1.55)	2407 (2006–2828)	1.11 (0.93–1.30)	-1.01 (-1.24 – -0.79)
Eastern Europe	2702 (2423–2982)	0.96 (0.86–1.06)	5126 (4364–5884)	1.50 (1.28–1.72)	1.15 (0.87–1.44)
**High income regions**					
Asia Pacific	2167 (1858–2484)	1.10 (0.94–1.27)	5858 (4615–7252)	1.24 (1.02–1.47)	0.35 (0.20–0.49)
North America	8251 (6952–9685)	2.27 (1.93–2.66)	4744 (3818–5783)	0.76 (0.62–0.91)	-4.25 (-4.53 – -3.96)
Western Europe	12860 (10920–14884)	2.15 (1.83–2.48)	8545 (6905–10425)	0.89 (0.74–1.07)	-3.39 (-3.65 – -3.13)
Australasia	658 (546–777)	2.71 (2.25–3.20)	347 (270–446)	0.63 (0.50–0.80)	-5.14 (-5.32 – -4.97)
**Latin America and Caribbean**					
Andean Latin America	49 (38–64)	0.24 (0.19–0.32)	117 (89–156)	0.20 (0.15–0.27)	-0.61 (-0.73 – -0.48)
Caribbean	325 (266–385)	1.28 (1.05–1.54)	403 (318–502)	0.75 (0.59–0.93)	-2.19 (-2.42 – -1.96)
Southern Latin America	792 (671–920)	1.69 (1.43–1.96)	716 (592–854)	0.83 (0.69–0.99)	-2.47 (-2.72 – -2.22)
Tropical Latin America	1485 (1312–1657)	1.60 (1.40–1.81)	3606 (2995–4329)	1.40 (1.16–1.68)	-0.86 (-1.08 – -0.65)
Central Latin America	391 (335–449)	0.48 (0.40–0.55)	733 (573–906)	0.29 (0.23–0.36)	-2.43 (-2.75 – -2.11)
**North Africa and Middle East**	523 (361–793)	0.30 (0.21–0.46)	1499 (1197–1830)	0.33 (0.26–0.40)	0.29 (0.21–0.38)
**South Asia**	1147 (670–2368)	0.22 (0.13–0.44)	4299 (2809–7207)	0.31 (0.20–0.52)	1.17 (1.10–1.24)
**South-East Asia, East Asia, and Oceania**					
East Asia	1306 (956–1776)	0.15 (0.11–0.20)	4623 (3421–6003)	0.22 (0.16–0.28)	1.58 (1.39–1.77)
Oceania	18 (12–25)	0.59 (0.41–0.83)	39 (27–53)	0.48 (0.35–0.66)	-0.85 (-0.95 – -0.75)
South-East Asia	759 (591–1050)	0.35 (0.26–0.49)	2374 (1903–2942)	0.41 (0.32–0.51)	0.27 (0.17–0.36)
**Sub-Saharan Africa**					
Central	90 (35–158)	0.40 (0.16–0.71)	192 (91–329)	0.34 (0.16–0.58)	-0.68 (-1.12 – -0.24)
Eastern	257 (109–454)	0.37 (0.16–0.65)	551 (243–941)	0.33 (0.15–0.56)	-0.75 (-1.00 – -0.50)
Southern	268 (208–335)	1.04 (0.78–1.34)	318 (257–382)	0.55 (0.44–0.66)	-2.73 (-3.04 – -2.42)
Western	224 (75–432)	0.27 (0.09–0.51)	498 (193–900)	0.25 (0.10–0.45)	-0.48 (-0.63 – -0.33)

ASMR: age-standardized mortality rate. EAPC: estimated annual percentage change. High SDI country number=48. High-middle SDI country number=51. Low SDI country number=34. Low-middle SDI country number=37. Middle SDI country number=34. UI: uncertainty interval. CI: confidence interval.

**Table 2:** DALYs and ASDR for aortic aneurysm due to smoking in 1990 and 2021, along with the EAPC from 1990 to 2021

*Location*	*DALYs 1990* *Number* *(95% UI)*	*ASDR 1990* *Rate/100000* *(95% UI)*	*DALYs 2021* *Number* *(95% UI)*	*ASDR 2021* *Rate/100000* *(95 % UI)*	*EAPC* *(95 % CI)*
**Global**	869302 (754922–1000697)	21.66 (18.72–24.96)	1157488 (989966–1325831)	13.35 (11.40–15.31)	-1.90 (-2.01 – -1.78)
**SDI Regions**					
High	496725 (433931–562690)	44.95 (39.30–50.75)	404881 (342182–473167)	21.73 (18.70–24.84)	-2.74 (-2.89 – -2.59)
High-middle	213842 (189699–239196)	20.77 (18.36–23.25)	344090 (298768–388951)	17.93 (15.57–20.26)	-0.82 (-1.00 – -0.64)
Middle	101670 (85288–124433)	9.02 (7.48–11.06)	244005 (203486–283082)	8.76 (7.29–10.19)	-0.40 (-0.52 – -0.29)
Low-middle	40520 (27295–67680)	6.22 (4.17–10.34)	125975 (97003–185689)	8.35 (6.40–12.44)	0.91 (0.86–0.95)
Low	15121 (6623–26705)	6.16 (2.73–10.83)	37038 (19207–61289)	6.55 (3.48–10.84)	0.04 (-0.15–0.24)
**Central Asia, Central and Eastern Europe**					
Central Asia	5049 (4308–6154)	9.93 (8.47–12.10)	15162 (12531–17839)	17.11 (14.18–20.14)	1.73 (1.52–1.95)
Central Europe	54802 (48776–60445)	36.37 (32.44–40.11)	58475 (49391–67864)	29.24 (24.89–33.71)	-1.02 (-1.24 – -0.79)
Eastern Europe	79512 (71995–86901)	28.49 (25.81–31.17)	141839 (121389–161203)	43.68 (37.66–49.23)	1.03 (0.72–1.34)
**High income regions**					
Asia Pacific	49329 (43274–55569)	24.19 (21.17–27.32)	113148 (94524–133714)	29.38 (25.23–33.62)	0.67 (0.54–0.79)
North America	180501 (156349–206337)	51.88 (45.19–59.01)	112814 (95731–131766)	20.06 (17.30–23.03)	-3.71 (-4.00 – -3.42)
Western Europe	266710 (233007–300531)	46.92 (41.33–52.58)	169368 (142587–199835)	20.36 (17.37–23.47)	-3.22 (-3.46 – -2.98)
Australasia	14314 (12222–16563)	59.64 (51.01–68.87)	7088 (5799–8713)	14.30 (11.88–17.22)	-5.04 (-5.22 – -4.86)
**Latin America and Caribbean**					
Andean Latin America	1340 (1065–1724)	6.05 (4.78–7.85)	3069 (2411–4021)	5.01 (3.93–6.58)	-0.57 (-0.70 – -0.43)
Caribbean	7264 (6121–8440)	27.72 (23.25–32.29)	8998 (7342–10931)	16.74 (13.66–20.33)	-2.05 (-2.30 – -1.81)
Southern Latin America	21053 (18191–23939)	44.60 (38.52–50.77)	18678 (15892–21570)	22.37 (19.16–25.70)	-2.42 (-2.66 – -2.19)
Tropical Latin America	43620 (39127–47860)	43.06 (38.34–47.56)	94971 (80972–110864)	36.07 (30.70–42.22)	-1.06 (-1.29 – -0.83)
Central Latin America	10836 (9409–12221)	11.99 (10.37–13.66)	19085 (15251–23377)	7.41 (5.91–9.07)	-2.42 (-2.74 – -2.10)
**North Africa and Middle East**	16410 (11251–25104)	8.38 (5.76–12.81)	43785 (35227–53485)	8.44 (6.75–10.28)	0.02 (-0.07–0.11)
**South Asia**	31760 (18663–65852)	5.17 (3.02–10.68)	107748 (69116–181259)	7.07 (4.58–11.91)	0.97 (0.92–1.02)
**South-East Asia, East Asia, and Oceania**					
East Asia	43507 (31708–59987)	4.31 (3.16–5.90)	137467 (101253–179382)	6.60 (4.84–8.58)	1.60 (1.40–1.80)
Oceania	573 (407–820)	16.11 (11.41–22.97)	1269 (897–1777)	13.57 (9.68–18.62)	-0.76 (-0.86 – -0.66)
South-East Asia	19001 (15005–25989)	7.39 (5.74–10.17)	57347 (46312–70455)	8.70 (7.04–10.78)	0.34 (0.24–0.43)
**Sub-Saharan Africa**					
Central	2669 (1045–4662)	10.47 (4.09–18.28)	6024 (2828–10440)	9.05 (4.26–15.50)	-0.57 (-0.99 – -0.15)
Eastern	7302 (3065–13204)	8.95 (3.79–15.92)	16657 (7242–28294)	8.33 (3.65–14.22)	-0.57 (-0.80 – -0.34)
Southern	7443 (6064–8880)	25.11 (19.98–30.53)	9469 (7720–11146)	14.49 (11.74–17.30)	-2.36 (-2.66 – -2.07)
Western	6307 (2073–12140)	6.60 (2.20–12.61)	15028 (5729–26825)	6.46 (2.50–11.57)	-0.39 (-0.56 – -0.23)

ASDR: age-standardized DALYs rate. EAPC: estimated annual percentage change. High SDI country number=48. High-middle SDI country number=51. Low SDI country number=34. Low-middle SDI country number=37. Middle SDI country number=34. UI: uncertainty interval. CI: confidence interval.

### Global burden of aortic aneurysm disease due to smoking factors

**Figure 2:**
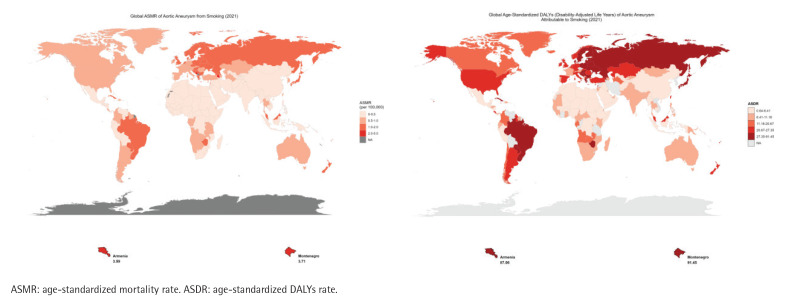
The disease burden of aortic aneurysm caused by smoking in 204 countries and regions: A) The ASMR in 2021; B) The ASDR in 2021

Smoking-related AA deaths increased in 180 countries worldwide during the study period, with ASMR rising in 76 countries. The absolute number of smoking-related AA deaths was highest in Japan, China, and the United States in 2021 (Supplementary file Table S1), but the countries with the highest ASMR were Armenia (3.59) and Montenegro (3.71); ASDR trends were similar to ASMR, and the highest countries were Armenia (87.06) and Montenegro (91.45) (Figure 2).

### Death and DALYs in different age groups worldwide in 2021

Supplementary file Figure S2 shows the distribution of AA deaths and disability-adjusted life years (DALYs) due to global smoking factors in 2021, stratified by age and sex. Overall, the number of AA deaths and DALYs caused by smoking factors increased first and then decreased with age in men and women in 2021. Most deaths in both sexes occurred in the 65–74 years age group, with a peak in the 70–74 years age group. DALYs occurred in the age group 60–69 years, with a peak in the 65–69 years group. ASMR and ASDR values increased first and then decreased with age in men, and increased with age in women (Supplementary file Figure S2).

### Global burden of gender-specific trends in AA from 1990 to 2021

The burden of aortic aneurysm associated with smoking showed significant differences between males and females. A sex-specific analysis of age-standardized mortality ratios (ASMR) and age-standardized DALYs rates (ASDR) showed a decreasing trend in disease burden in both sexes, but the point estimates for the burden remained greater in men than in women (Supplementary file Figure S3). Among them, Central Asia, East Asia, and South Asia are areas with a high prevalence of aortic aneurysm disease burden caused by smoking, and the point estimates for the male burden show an increasing trend and remain greater than those for females (Supplementary file Figures S4–S6).

### ASMR and ASDR for predicting AA caused by smoking in the next 15 years

According to the ARIMA model, ASMR and ASDR of AA caused by smoking will continue to decline worldwide over the next 15 years. Meanwhile, ASMR and ASDR of AA caused by smoking in Central Asia, East Asia and South Asia will gradually rise, and ASMR and ASDR changes in Eastern Europe tend to stabilize (Supplementary file Figure S7).

### Correlation analysis between SDI and ASMR/ASDR

**Figure 3:**
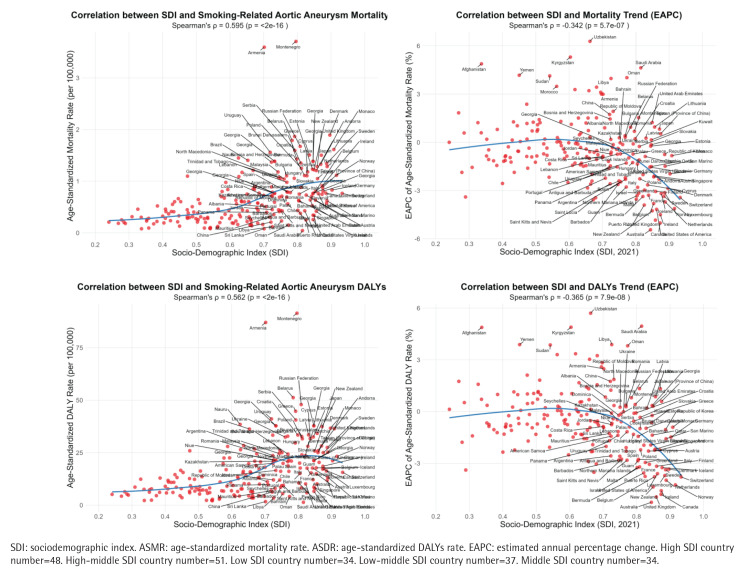
The relationship between SDI and relevant indicators of aortic aneurysm due to smoking in each country: A) The relationship between SDI and ASMR in 2021; B) The relationship between SDI and ASDR in 2021; C) The EAPC relationship between SDI and ASMR from 1990 to 2021; D) The EAPC relationship between SDI and ASDR from 1990 to 2021

In 2021, there was a positive relationship between SDI and ASMR (R=0.595, p<0.001) and ASDR (R=0.562, p<0.001) associated with smoking-related AA in each country (Figures 3A and 3B). However, SDI showed a negative relationship with annual trends in smoking-related AA burden [i.e. ASMR estimated annual percent change (EAPC) and ASDR (EAPC)] across countries between 1990 and 2021 (Figures 3C and 3D).

### Frontier analysis of the burden of AA attributed to smoking

**Figure 4:**
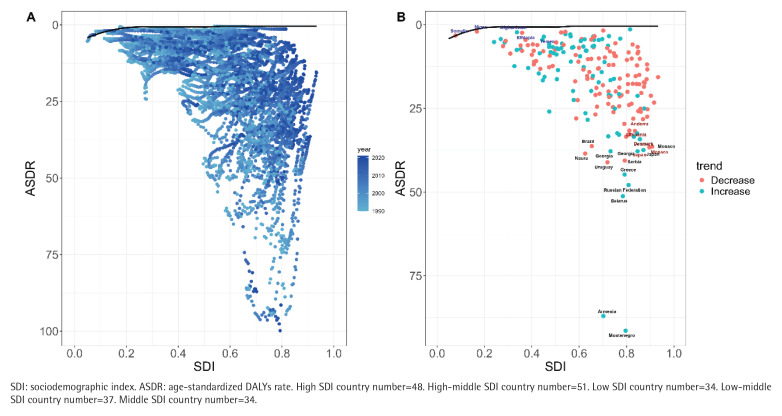
Frontier analysis of different global SDI levels and smoking-related aortic aneurysm from 1990 to 2021: A) Frontier analysis of temporal trends in ASDR from 1990 to 2021, with color gradients indicating the year course (light blue to dark blue). The front line is marked in black and represents the best achievable performance at each SDI level; B) Frontier analysis 2021 ASDR relative to Frontier analysis curve status in each country. The color of punctate marks indicates their direction of change since 1990 (blue: rise; red: fall). Labels identify countries furthest from the front line (red) and closest to the front line (blue), representing the least and most efficient countries, respectively. All rates are expressed per 100000 population

With frontier analysis, this study explores potential optimal scenarios for disease burden management across countries and regions at their specific sociodemographic index (SDI) level. In terms of ASDR indicators, five low SDI countries, Afghanistan, Niger, Ethiopia, Somalia and Yemen, were closest to the front line of efficiency and emerged as relatively excellent cases in resource-limited conditions. In contrast, five high SDI countries, Andorra, Lithuania, Denmark, Monaco and Japan, are furthest from the front line and represent cases of abnormal performance at high levels of development. In addition, countries such as Uruguay, Greece, the Russian Federation, Belarus, Armenia and Montenegro also have significant gaps from the former line, showing a large potential room for improvement (Figure 4).

### Transnational health inequality analysis

In 204 countries, there were significant absolute and relative differences between ASMR and ASDR and SDI due to aortic aneurysm caused by smoking factors. This inequality is particularly evident in countries with high SDI: the ASMR’s inequality slope index (SII) rose from 1.4 in 1990 to 1.54 in 2021, highlighting the increasing positive association between SDI and aortic aneurysm burden due to smoking factors. The inequality slope index (SII) of ASDR decreased from 36.88 in 1990 to 34.28 in 2021, indicating that the positive relationship between SDI and aortic aneurysm burden caused by smoking factors weakened. However, the results of the concentration curves indicate an improvement in relative inequalities between countries. The concentration index decreased from 0.58 (95% UI: 0.69–0.47) in 1990 to 0.36 (95% UI: 0.23–0.49) in 2021, and the concentration curve in 2021 more approached the absolute equalization line (Supplementary file Figure S8).

### Decomposition analysis of aortic aneurysm disease burden associated with smoking in different SDI regions worldwide from 1990 to 2021

**Figure 5:**
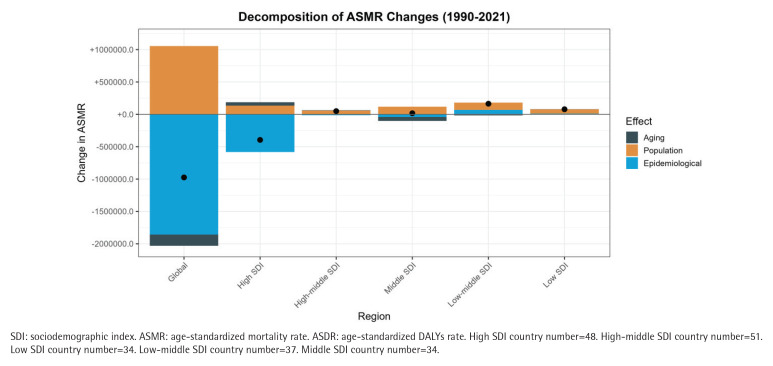
Decomposition analysis of changes in aortic aneurysm disease burden caused by smoking worldwide and in different SDI regions from 1990 to 2021: A) ASMR; B) ASDR. Black dots represent the final change values due to a combination of three factors: population growth, population aging, and changes in epidemiological trends

We investigated the impact of population growth, population ageing, and changing epidemiological trends on smoking-related burden of aortic aneurysm disease (Table 3). Decomposition analysis showed that the decline of ASMR and ASDR in smoking-related aortic aneurysm diseases was mainly driven by changes in epidemiological trends worldwide in areas with high SDI. It is worth noting that changes in population growth trends have positively contributed to both ASMR and ASDR in the overall population worldwide and in areas with high SDI, but this has not changed the overall downward trend. In addition to high SDI areas, the rise in ASMR and ASDR associated with smoking-related aortic aneurysm disease in other SDI areas was mainly driven by changes in population growth trends (Figure 5).

**Table 3:** Effect of aging population, population growth and epidemiological trends on changes in ASMR and ASDR of smoking-related aortic aneurysm in different SDI areas worldwide from 1990 to 2021

*SDI Regions*	*Measure*	*Total* *difference*	*Aging* *effect*	*Population* *effect*	*Rate* *effect*	*Aging* *percent*	*Population* *percent*	*Rate* *percent*
**High**	ASMR	-76678.91	1917.24	35898.32	-114494.48	-2.50	-46.82	149.32
	ASDR	-1236935.57	1771.42	521217.24	-1759924.23	-0.14	-42.14	142.28
**High-middle**	ASMR	-4710.60	713.44	15826.54	-21250.58	-15.15	-335.98	451.12
	ASDR	-18257.89	2246.74	267,717.96	-288222.59	-12.31	-1466.31	1578.62
**Middle**	ASMR	18903.75	2072.49	27256.18	-10424.92	10.96	144.18	-55.15
	ASDR	330086.73	17451.50	418747.17	-106111.94	5.29	126.86	-32.15
**Low-middle**	ASMR	43291.96	208.70	23242.73	19840.54	0.48	53.69	45.83
	ASDR	605047.00	18293.43	340482.01	246271.56	3.02	56.27	40.70
**Low**	ASMR	19362.80	-795.10	16915.06	3242.83	-4.11	87.36	16.75
	ASDR	289310.10	-4901.95	247429.24	46782.82	-1.69	85.52	16.17

High SDI country number=48. High-middle SDI country number=51. Low SDI country number=34. Low-middle SDI country number=37. Middle SDI country number=34.

## DISCUSSION

This study provides a comprehensive analysis of the global burden of smoking-related AA, leveraging the robust and standardized methodology of the GBD study. Our findings, based on data from 1990 to 2021, reveal that despite an overall increase in the absolute number of deaths, the ASMR and ASDR attributable to smoking showed a significant declining trend. This overall improvement was primarily driven by favorable changes in epidemiological trends, as indicated by our decomposition analysis.

The data showed that the high SDI region accounted for 40.3% of aortic aneurysm-related deaths worldwide in 2021, and its ASMR was the highest, which is consistent with the existing literature conclusion that the high SDI region continues to bear the heaviest aortic aneurysm burden due to the aging population and high comorbidity of atherosclerosis^18,19^. While ASMR and ASDR have the highest decreasing trend in high SDI areas, one of the reasons for this may be better control of risk factors in high SDI areas^20^. In sharp contrast, ASMR and ASDR in low-middle SDI areas such as Central Asia, East Asia, South Asia, and Eastern Europe are on the rise, and their disease burden is increasing significantly, possibly due to increased smoking prevalence and limited medical resources^18^. This study finds that the burden of disease from smoking-related aortic aneurysms is particularly significant in older age groups worldwide. Specifically, global data from 2021 suggests that deaths and DALYs of smoking-related aortic aneurysms in men peak in the 65–74 years age group, which is consistent with the natural history of aortic aneurysms, that aneurysms expand with age and increase the risk of rupture^21^. In women, ASMR and ASDR rise with age, and possible mechanisms include diminished estrogen protection of the vessel wall (increased risk in post-menopausal women), or accelerated late progression due to delayed diagnosis in women, which requires further validation. This is consistent with the available literature, where multiple studies have demonstrated that smoking is a major risk factor for aortic aneurysms and that a high prevalence is common in men and in older age groups older than 65 years. For example, a systematic review noted a high incidence of aortic aneurysms in smokers and recommended screening for male smokers aged 65–75 years, which is consistent with the peak mortality age group in this study (70–74 years), suggesting that global data support targeted screening strategies^22^. Another Mendelian randomization study provides further causal evidence that smoking directly increases aortic aneurysm risk and highlights co-factors such as hypertension and obesity, which are consistent with the increasing trend in age-related burden in this study, as older age groups often have additional cardiovascular risks^23^.

Smoking-related AA burden remains consistently higher in men than in women, well above the global average. This is consistent with previous studies, which have shown that smokers have a significantly higher relative risk of aortic aneurysm than other smoking-related diseases, and that men have a 2.5-fold higher risk than women^24,25^. The Copenhagen Demographic Study found that smoking was the strongest predictor of aortic aneurysm, with a significantly higher risk in men than in women. It is important to note that Central Asia, East Asia and South Asia are highly prevalent regions, where the burden of disease in men is increasing and continues to be higher than in women. These results highlight the central role of smoking in driving aortic aneurysm burden, particularly in Asian male populations. This has important implications for public health practice and future research, such as enhancing smoking cessation interventions and promoting ultrasound screening for male populations in high incidence areas (Central Asia, East Asia, and South Asia)^22^. Policy, tobacco control policies need to be prioritized in smoking-endemic areas to mitigate the rising burden. We also call for global collaboration to expand gender-specific intervention strategies.

According to predictions from the ARIMA model, smoking is established as a core risk factor for aortic aneurysm, and its ASMR and ASDR worldwide are expected to show a decreasing trend over the next 15 years. These projections were generated using an ARIMA model, a well-established statistical tool for time-series analysis^23^. The model’s ability to capture underlying trends and cyclical patterns provides a scientifically robust basis for these forecasts, thereby offering valuable insights for long-term public health planning and resource allocation. This observed decline may be partly attributed to the strengthening of global smoking control policies and health interventions in the context of an aging population. However, regional heterogeneity is significant: ASMR and ASDR have risen in Central Asia, East Asia, and South Asia and may be associated with high smoking rates, inadequate health care resources, and socioeconomic factors in these regions^24,25^; while changes in Eastern Europe have stabilized, a pattern which may reflect the sustaining effects of their existing public health systems. The prediction results of this study are generally consistent with the existing literature results, but there are differences at the regional level. First, the prediction of a global decline in ASDR is in line with the GBD study. For example, 1990–2021 data analysis found a 26.8% reduction in aortic aneurysm ASDR, mainly due to smoking control and prevention strategies^25^. However, another systematic analysis found that the burden of premature aortic aneurysm increased in areas with low SDI, particularly in Central Asia, East Asia, and South Asia, where smoking prevalence was higher, resulting in increased burden^24^, and the regional rise predicted in this study (particularly in Asia) reinforced the view of this systematic analysis^24^. Therefore, areas with low SDI (e.g. South Asia) should prioritize the strengthening of medical infrastructure and implement targeted smoking cessation interventions and screening programs^25^.

As far as the SDI is concerned, high SDI areas showed higher ASMR and ASDR, which may reflect amplification of smoking exposure and disease development by socio-economic factors such as healthcare resource allocation and lifestyle habits^26^. At the same time, the negative trend from 1990 to 2021 showed that although countries with high SDI had a heavy initial burden, their burden showed a rapid decline through public health interventions (e.g. tobacco control policies)^27^; while countries with low SDI had an increased burden, highlighting the imbalance in global prevention and control^2,26^, suggesting that countries with low SDI need to strengthen tobacco hazard education and early screening^22,25^. In addition, given that patients with aortic aneurysms present a higher risk of cancer than patients without aortic aneurysms, we recommend that smoking-related aortic aneurysms be included in multimorbid management programs to promote integrated cardiovascular and oncologic prevention studies^28^.

In the frontier analysis, we found that some low SDI countries (e.g. Afghanistan, Niger) were closer to the front line of efficiency in ASDR, indicating that they achieved relatively optimized disease management with limited resources; excellent performance in countries such as Ethiopia may stem from targeted interventions (e.g. primary care focuses on high-risk groups) and are in line with strategies to ‘prioritize the control of interventionable risk factors with limited resources’^29,30^. Countries with high SDI (e.g. Japan, Denmark), on the other hand, deviate significantly from the front line, suggesting that their existing healthcare system fails to effectively control tobacco-related aortic aneurysm burden^29,31^. Notably, there is significant room for improvement in countries with high SDI such as Uruguay and the Russian Federation, which may be associated with differences in tobacco control policy implementation^32^. The inefficiency in Japan and other countries may be related to the high proportion of the elderly population and insufficient compliance with screening. The literature states that male smokers over 65 years of age should undergo ultrasound screening, but the actual screening rate is less than 30%^22,33^.

Health inequality analysis showed improvements in health disparities in smoking-related aortic aneurysm disease burden between 1990 and 2021. Although the disease burden gap between low and high SDI areas has narrowed, suggesting that wealth gaps may be narrowing in some areas, absolute inequalities in smoking-related aortic aneurysm burden persist worldwide, particularly highlighting differences in healthcare resource allocation in SDI stratification^22,29^. In addition, the inequality slope index (SII) of ASMR in countries with high SDI has risen, indicating that there is an increasing positive correlation between SDI and aortic aneurysm burden caused by smoking factors, warning us that social progress has not equally translated into disease prevention and control benefits. Comprehensive smoking cessation support with targeted screening is the core path to reduce the burden of future disease^32^.

### Limitations

This study has several limitations that should be considered. First, the analysis relies on secondary data sources such as death registries and health surveys, which may involve underestimation or reporting bias, particularly in high-incidence Asian regions with limited data quality. The population attributable fraction (PAF) for smoking was derived from meta-analyses and prevalence estimates, which may not fully reflect regional variations or specific smoking behaviors. Second, not all potential confounders were adjusted for; for example, synergistic effects between risk factors such as hypertension and smoking were not accounted for, and residual confounding by unmeasured factors remains possible. Third, future projections to 2036 were generated using the ARIMA model based on historical trends, and thus do not incorporate potential impacts of future policy interventions, technological advances (e.g. novel early screening tools), shifts in treatment thresholds, or major tobacco control policies. Fourth, as an ecological and population-level study, the observed associations do not imply individual-level causation. Finally, multiple comparisons were conducted without statistical correction, which may increase Type I error, and the assessment of smoking exposure did not include emerging products such as e-cigarettes.

## CONCLUSIONS

This study reveals the current status of global health inequalities by systematically analyzing the burden of aortic aneurysm disease caused by smoking, and may provide a scientific basis for formulating public health policies for different regions and development levels. Although, the burden of aortic aneurysms due to smoking is decreasing worldwide, health inequalities remain significant across SDI regions, and effective measures are urgently needed to narrow this gap. Our findings underscore the continuing need for effective tobacco control and targeted screening strategies to mitigate the global burden of smoking-related AA, particularly in regions where the burden is projected to rise.

## Supplementary Material



## Data Availability

The data supporting this research are available from the following publicly accessible source: The Global Health Data Exchange (GHDx) platform (https://vizhub.healthdata.org/gbd-results/). Additional data underlying this study are available from the corresponding author upon reasonable request.
