# Supplementary material for: The global burden of aortic aneurysm attributable to smoking from 1990 to 2021: Current trends and projections for 2036
Source: Tob Induc Dis. 2026 Feb 17;24:10.18332/tid/215179. doi: 10.18332/tid/215179 (PMC12914652; doi:10.18332/tid/215179)
Supplement: Supplementary file 1 [file TID-24-23-s1.pdf]

The Global Burden of Aortic Aneurysm Attributable to Smoking from 1990 to 2021: Current Trends and Projections for 2036

Supplementary data

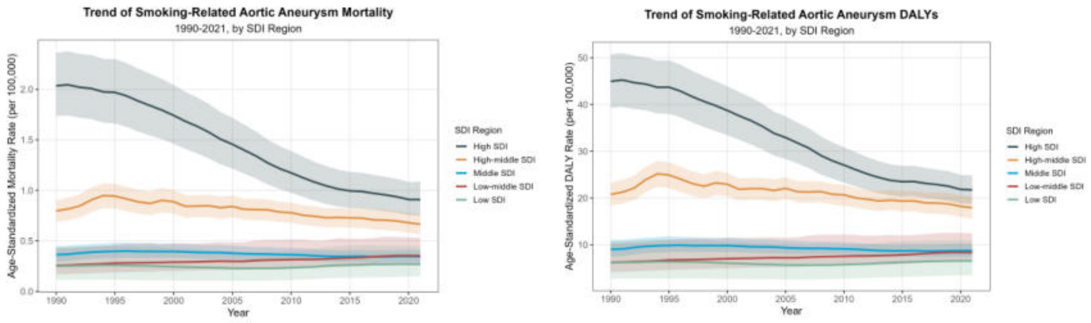

Figure S1. Trends of ASDR and ASMR for smoking-related aortic aneurysm by across diverse SDI region from 1990 to 2021. Notes: ASMR, Age-standardized mortality rate; ASDR, Age-standardized DALY rate, SDI, Socio-demographic index. Notes: High SDI country number = 48; High-middle SDI country number = 51; Low SDI country number = 34; Low-middle SDI country number = 37; Middle SDI country number = 34.

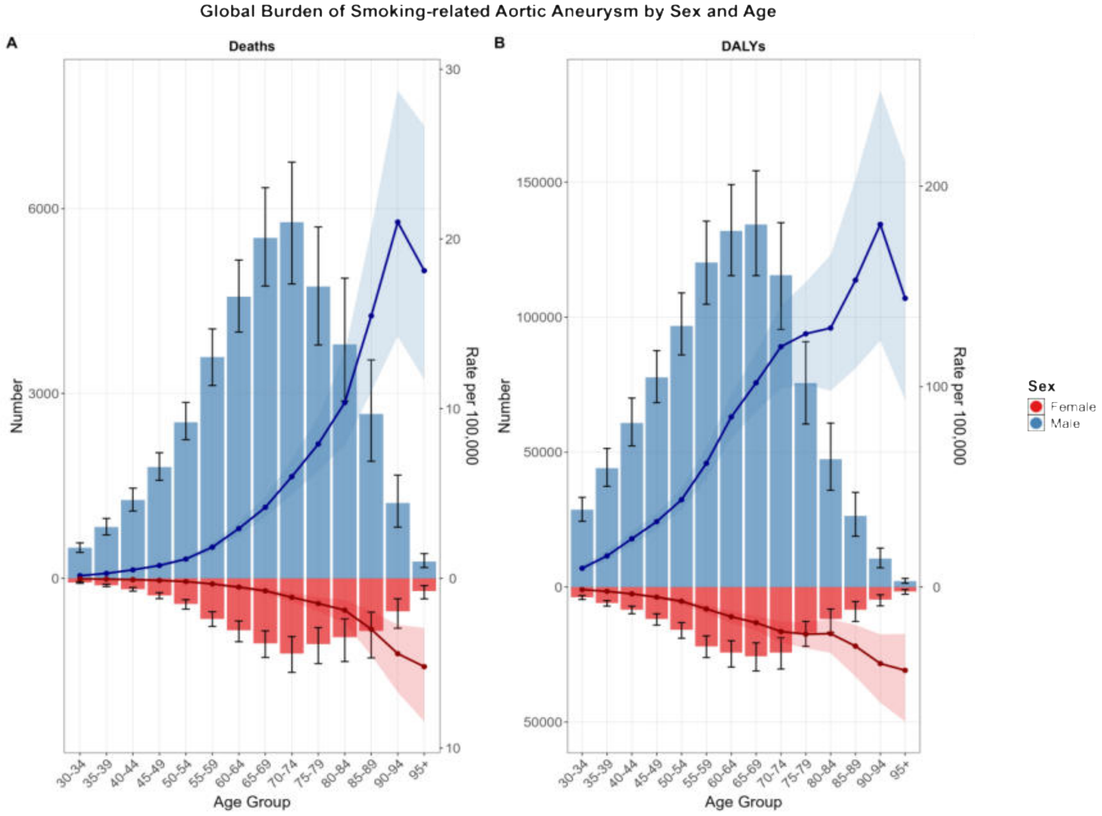

Figure S2. Age-specific number and rates of DALYs and deaths due to aortic aneurysm attributable to smoking in 2021. Shaded areas represent 95% uncertainty intervals (UI). Notes: DALYs, Disability-adjusted life years.

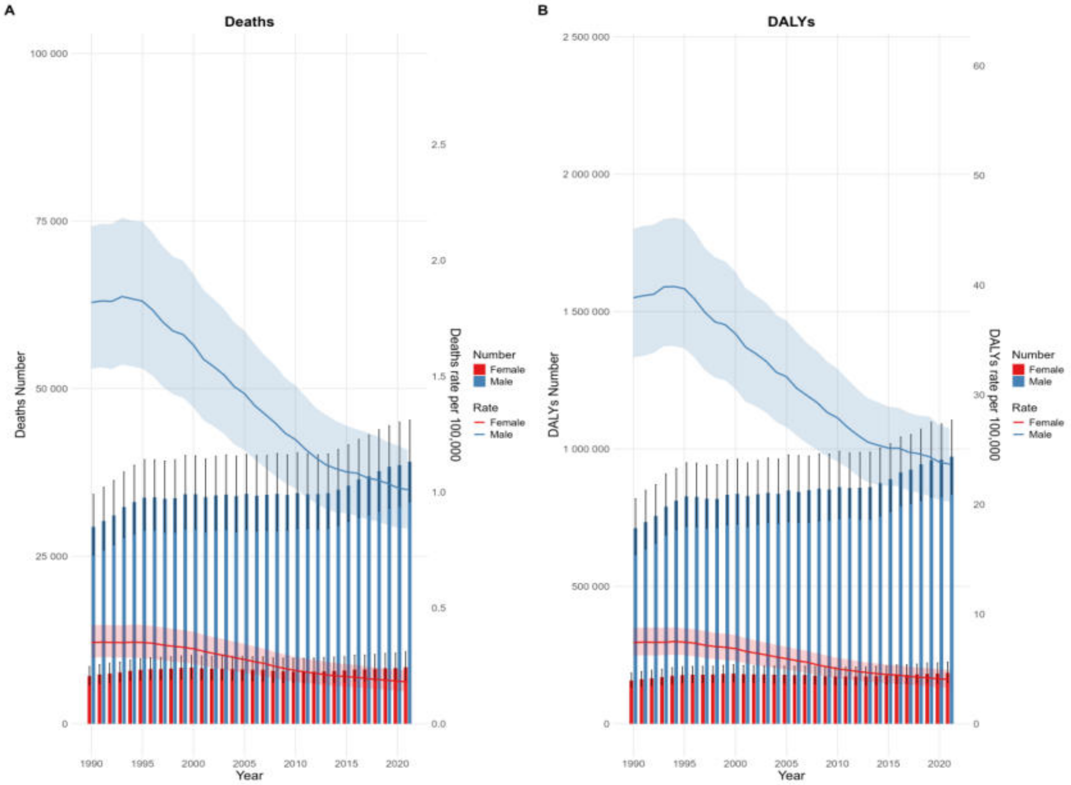

Figure S3. Global time trends in aortic aneurysm deaths and DALYs due to smoking by year from 1990 to 2021. (A) number of deaths versus ASMR; (B) number of DALYs versus ASDR. Error bars and shaded areas denote 95% uncertainty intervals (UI). Notes: DALYs, Disability-adjusted life years; ASMR, Age-standardized mortality rate; ASDR, Age-standardized DALY rate.

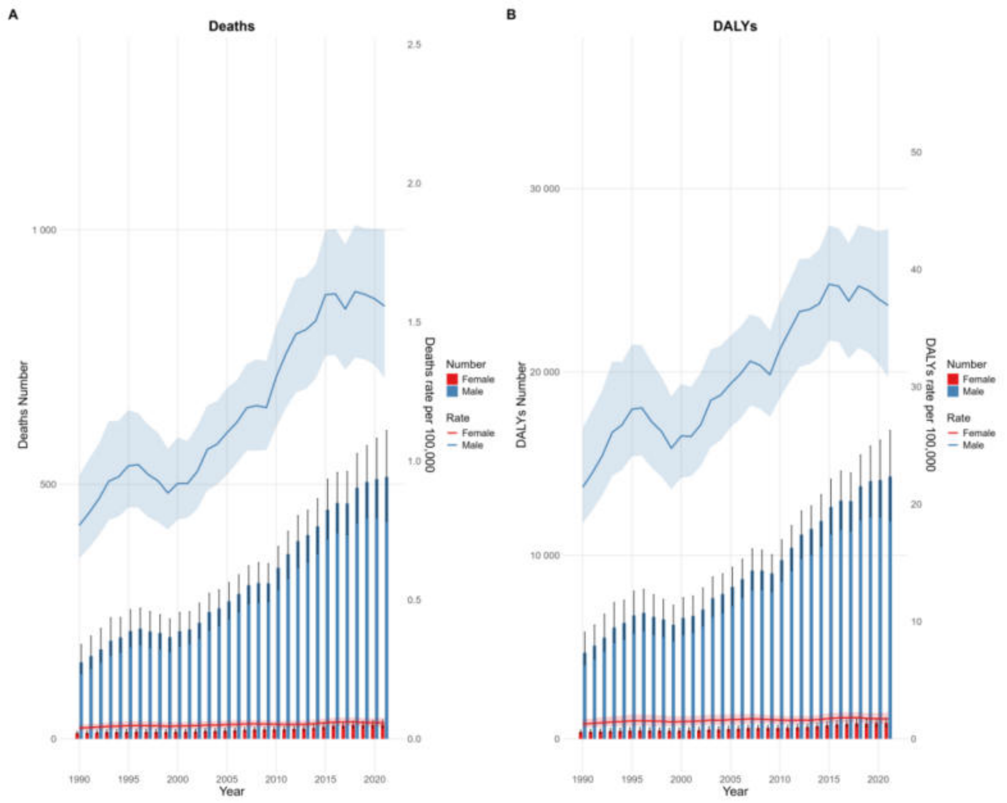

Figure S4. Timing trend of aortic aneurysm deaths and DALYs due to smoking by year in Central Asia, 1990 – 2021. Error bars and shaded areas denote 95% uncertainty intervals (UI). Notes: DALYs, Disability-adjusted life years.

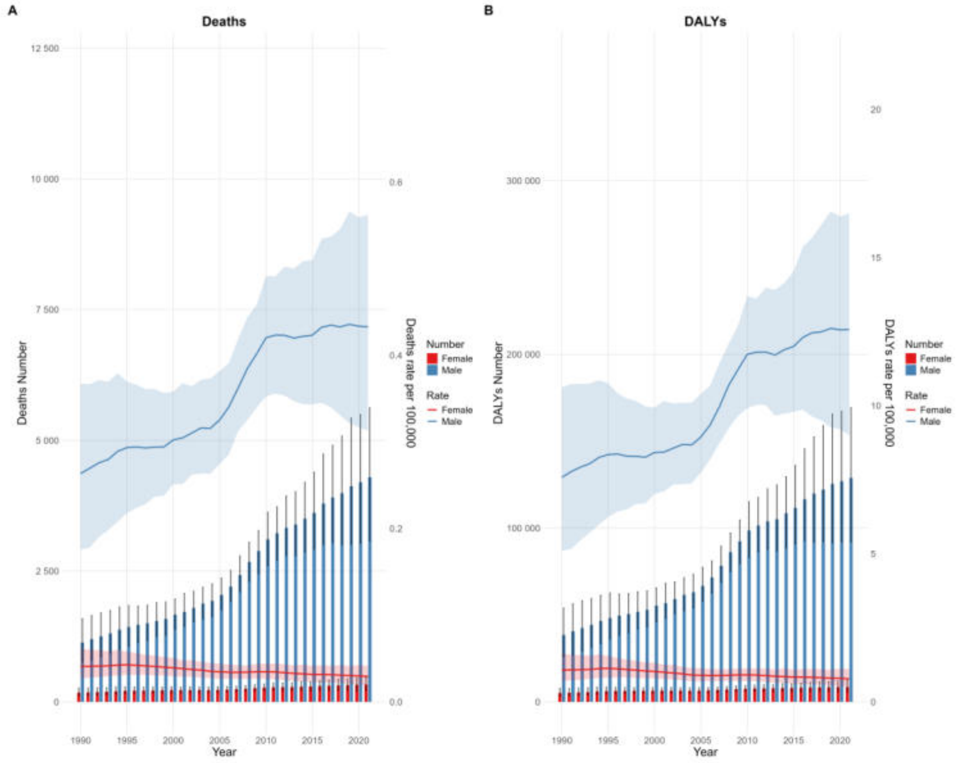

Figure S5 Timing trend of aortic aneurysm deaths and DALYs due to smoking by year in East Asia, 1990 – 2021. Error bars and shaded areas denote 95% uncertainty intervals (UI). Notes: DALYs, Disability-adjusted life years.

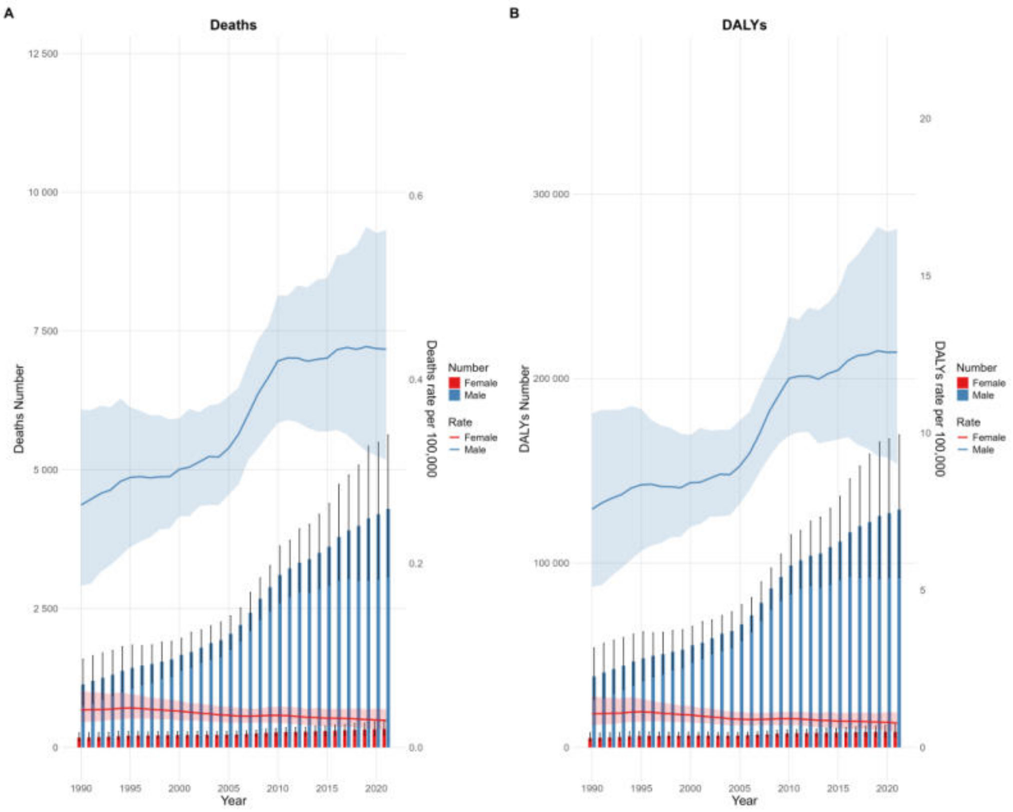

Figure S6. Timing trend of AA deaths and DALYs due to smoking by year in South Asia, 1990 – 2021. Error bars and shaded areas denote 95% uncertainty intervals (UI). Notes: DALYs，Disability-adjusted life years

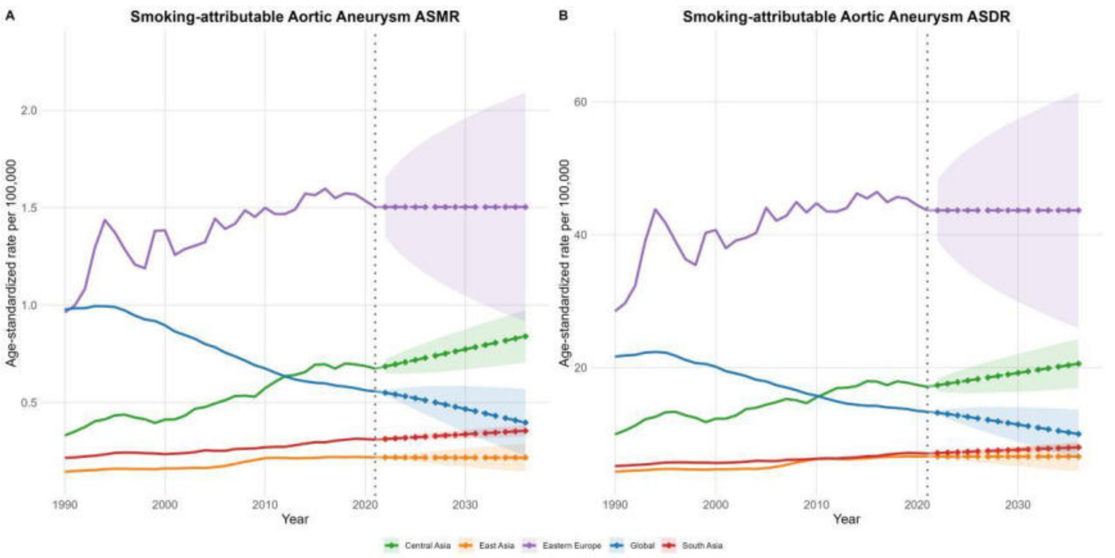

Figure S7. Temporal trend of ASMR and ASDR in areas with high incidence of AA caused by smoking worldwide from 1990 to 2036. Different colors represent different regions, dashed lines represent predicted trends and shaded areas around them indicate 95% CIs for this predicted trend. Notes: ASMR，Age-standardized mortality rate；ASDR，Age-standardized DALY rate；CI，Confidence interval

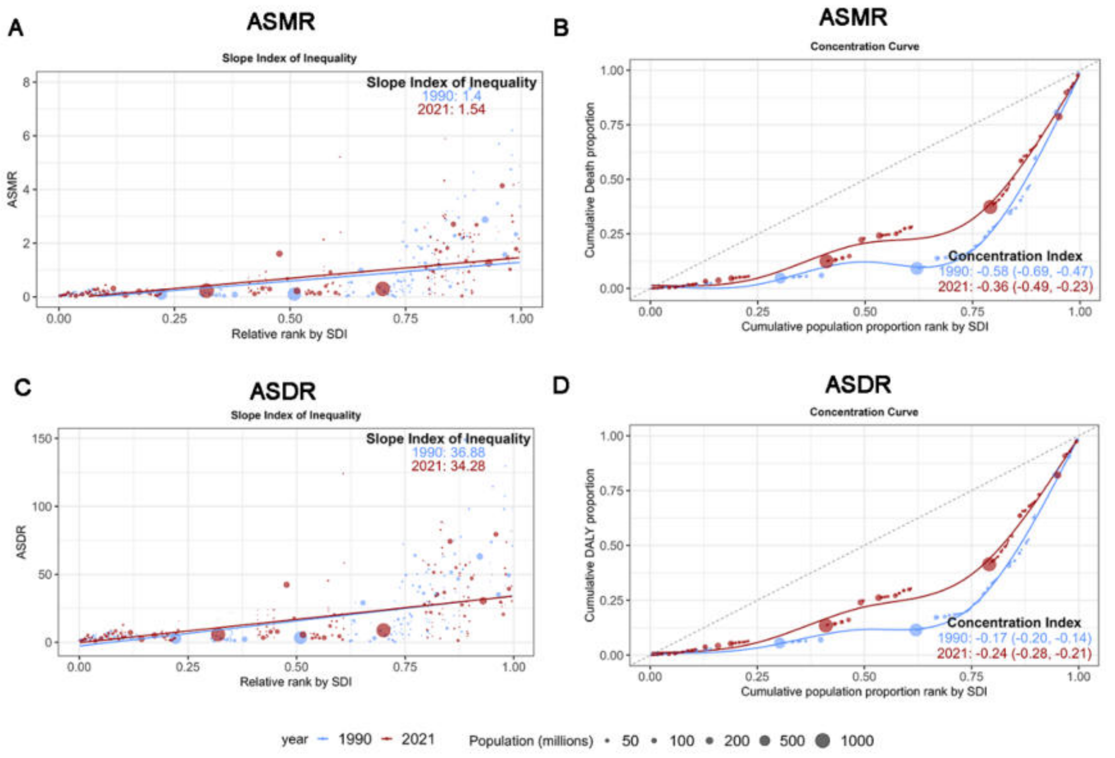

Figure S8. Concentrated curves of health inequalities associated with SDI for smoking-related AA worldwide in 1990 and 2021. Global smoking-related AA burden ASMR health inequality regression curve (A) and concentration curve (B); global smoking-related aortic aneurysm ASDR health inequality regression curve (C)

and concentration curve (D). Notes:SDI，Socio-demographic index；ASMR，Age-standardized mortality rate；ASDR，Age-standardized DALY rate. High SDI country number：48；High-middle SDI country number：51；Low SDI country number：34；Low-middle SDI country number：37；Middle SDI country number：34.

Table S1. The absolute number of smoking-related aortic aneurysm deaths and ASMR in 1990 and 2021, and the EAPC in 204 countries.

Country	Death_1990	ASMR_1990	Death_2021	ASMR_2021	EAPC
Japan	1,977 (1,706-2,256)	1.17 (1.01-1.34)	5,289 (4,116-6,579)	1.50 (1.25-1.78)	0.82 (0.72-0.93)
China	1,179 (839-1,634)	0.14 (0.10-0.19)	4,202 (3,023-5,569)	0.21 (0.15-0.27)	1.73 (1.47-1.99)
United States of America	7,302 (6,131-8,547)	2.22 (1.87-2.58)	4,200 (3,373-5,108)	0.76 (0.62-0.91)	-4.17 (-4.45--3.88)
Russian Federation	1,780 (1,585-1,950)	0.98 (0.87-1.07)	3,918 (3,293-4,530)	1.67 (1.41-1.92)	1.53 (1.18-1.87)
Brazil	1,463 (1,294-1,631)	1.62 (1.41-1.83)	3,539 (2,937-4,243)	1.40 (1.16-1.69)	-0.89 (-1.11--0.67)
India	856 (485-1,808)	0.20 (0.11-0.42)	3,274 (1,998-5,539)	0.29 (0.18-0.49)	1.30 (1.17-1.44)
United Kingdom	4,443 (3,746-5,166)	4.57 (3.87-5.29)	1,819 (1,384-2,293)	1.28 (1.00-1.59)	-4.84 (-5.22--4.46)
Germany	1,865 (1,554-2,188)	1.42 (1.20-1.66)	1,523 (1,241-1,877)	0.83 (0.69-0.99)	-1.96 (-2.13--1.79)
Italy	1,294 (1,122-1,474)	1.44 (1.25-1.63)	1,085 (873-1,325)	0.75 (0.62-0.88)	-2.80 (-3.15--2.45)
Poland	949 (842-1,064)	2.16 (1.91-2.42)	890 (726-1,070)	1.25 (1.03-1.49)	-2.27 (-2.55--1.99)
France	1,259 (1,047-1,482)	1.49 (1.26-1.74)	888 (703-1,101)	0.64 (0.52-0.77)	-3.29 (-3.59--3.00)
Turkey	371 (249-570)	1.02 (0.70-1.58)	866 (643-1,144)	0.91 (0.67-1.20)	-0.58 (-0.79--0.37)
Spain	699 (604-791)	1.27 (1.10-1.4	765 (624-925)	0.79 (0.66-0.9	-2.30 (-2.67--1.9

		3)		4)	3)
Ukraine	663 (560-778)	0.93 (0.79-1.09)	756 (484-1,056)	1.04 (0.67-1.46)	-0.14 (-0.61-0.33)
Indonesia	158 (104-237)	0.18 (0.12-0.28)	615 (348-909)	0.31 (0.18-0.47)	1.51 (1.37-1.66)
Thailand	200 (142-296)	0.68 (0.47-1.01)	611 (442-838)	0.57 (0.42-0.79)	-1.07 (-1.25--0.89)
Canada	947 (787-1,130)	2.86 (2.38-3.40)	543 (431-680)	0.75 (0.60-0.92)	-5.02 (-5.33--4.72)
Republic of Korea	170 (101-254)	0.65 (0.39-0.97)	528 (394-668)	0.57 (0.43-0.72)	-0.76 (-1.04--0.47)
Bangladesh	119 (55-282)	0.27 (0.13-0.65)	510 (298-1,012)	0.40 (0.23-0.80)	1.25 (1.08-1.41)
Argentina	623 (522-732)	1.90 (1.59-2.23)	492 (402-589)	0.89 (0.73-1.06)	-2.58 (-2.83--2.34)
Netherlands	708 (597-831)	3.44 (2.92-4.02)	481 (379-605)	1.28 (1.02-1.59)	-3.71 (-4.11--3.30)
Greece	297 (259-340)	1.95 (1.71-2.23)	441 (368-524)	1.87 (1.59-2.18)	-0.73 (-1.04--0.42)
Pakistan	150 (87-269)	0.28 (0.16-0.51)	428 (275-688)	0.38 (0.25-0.60)	0.69 (0.37-1.02)
Taiwan (Province of China)	92 (81-104)	0.57 (0.50-0.65)	354 (297-411)	0.88 (0.74-1.01)	0.53 (-0.10-1.16)
Colombia	177 (151-208)	0.98 (0.83-1.16)	333 (249-435)	0.60 (0.45-0.79)	-2.84 (-3.30--2.38)
Philippines	122 (98-150)	0.48 (0.38-0.59)	328 (252-421)	0.42 (0.32-0.54)	-0.42 (-0.53--0.32)
Viet Nam	83 (57-134)	0.22 (0.15-0.35)	321 (213-464)	0.35 (0.23-0.51)	1.51 (1.42-1.61)
Sweden	490	3.08	313	1.34	-3.14

	(397-580)	(2.54-3.62)	(239-400)	(1.04-1.70)	(-3.50--2.78)
Malaysia	100 (71-133)	1.19 (0.85-1.61)	304 (231-392)	1.18 (0.89-1.53)	-0.44 (-0.80--0.08)
Belarus	152 (128-195)	1.18 (1.00-1.51)	281 (219-353)	1.79 (1.40-2.25)	0.94 (0.50-1.38)
Serbia	180 (138-227)	1.69 (1.27-2.17)	261 (197-345)	1.59 (1.21-2.08)	-0.10 (-0.35-0.14)
Denmark	320 (276-364)	3.81 (3.30-4.31)	244 (194-298)	1.89 (1.54-2.28)	-2.86 (-3.28--2.44)
Australia	499 (415-594)	2.47 (2.05-2.94)	241 (182-315)	0.53 (0.41-0.67)	-5.44 (-5.62--5.25)
Cuba	224 (184-266)	2.19 (1.80-2.60)	240 (188-301)	1.21 (0.95-1.51)	-2.41 (-2.70--2.13)
Czechia	194 (167-225)	1.40 (1.22-1.63)	234 (187-288)	1.11 (0.90-1.36)	-0.88 (-1.27--0.49)
Romania	147 (126-167)	0.53 (0.46-0.61)	232 (186-280)	0.69 (0.55-0.83)	0.32 (0.07-0.58)
South Africa	221 (166-277)	1.10 (0.80-1.41)	226 (178-278)	0.48 (0.37-0.59)	-3.45 (-3.79--3.11)
Belgium	374 (314-440)	2.36 (2.00-2.74)	194 (153-237)	0.80 (0.65-0.96)	-3.81 (-4.07--3.56)
Hungary	197 (169-229)	1.36 (1.16-1.56)	192 (155-232)	1.06 (0.87-1.26)	-1.26 (-1.45--1.06)
Nigeria	102 (32-214)	0.25 (0.08-0.50)	190 (64-394)	0.21 (0.07-0.43)	-0.85 (-1.04--0.66)
Mexico	104 (88-119)	0.27 (0.22-0.31)	171 (132-215)	0.14 (0.11-0.17)	-2.60 (-2.84--2.36)
United Republic of Tanzania	66 (30-114)	0.65 (0.30-1.15)	167 (70-298)	0.66 (0.28-1.18)	-0.55 (-0.80--0.30)

Armenia	46 (38-57)	1.64 (1.34-2.06)	156 (124-190)	3.59 (2.85-4.38)	2.99 (2.51-3.46)
Switzerland	232 (192-271)	2.17 (1.82-2.52)	150 (116-188)	0.76 (0.60-0.94)	-3.43 (-3.59--3.27)
Bulgaria	109 (92-129)	0.92 (0.77-1.08)	143 (106-182)	1.10 (0.82-1.41)	0.41 (0.08-0.74)
Chile	81 (68-94)	0.79 (0.66-0.93)	142 (115-167)	0.56 (0.46-0.66)	-1.29 (-1.65--0.92)
Kazakhstan	69 (56-91)	0.51 (0.41-0.68)	136 (105-177)	0.71 (0.55-0.92)	0.18 (-0.34-0.70)
Venezuela (Bolivarian Republic of)	73 (61-85)	0.76 (0.63-0.90)	131 (92-179)	0.44 (0.31-0.60)	-2.50 (-2.85--2.15)
Croatia	79 (66-95)	1.34 (1.11-1.60)	129 (100-162)	1.43 (1.11-1.78)	0.22 (-0.07-0.50)
Finland	200 (164-239)	2.79 (2.31-3.32)	129 (99-162)	1.05 (0.84-1.28)	-3.06 (-3.38--2.74)
Iran (Islamic Republic of)	23 (16-33)	0.09 (0.06-0.12)	128 (101-156)	0.16 (0.13-0.20)	3.00 (2.58-3.42)
Myanmar	69 (38-111)	0.35 (0.20-0.55)	121 (86-171)	0.28 (0.20-0.40)	-0.81 (-0.90--0.72)
Egypt	37 (23-62)	0.14 (0.08-0.23)	117 (91-149)	0.19 (0.15-0.24)	1.12 (1.03-1.21)
Norway	224 (186-265)	3.12 (2.62-3.64)	117 (90-151)	1.11 (0.87-1.41)	-4.12 (-4.57--3.66)
Austria	138 (116-162)	1.17 (1.00-1.37)	113 (91-138)	0.63 (0.52-0.76)	-2.15 (-2.35--1.95)
New Zealand	159 (129-191)	3.91 (3.19-4.67)	106 (83-133)	1.22 (0.96-1.51)	-4.26 (-4.46--4.06)
Georgia	82 (69-96)	1.16 (0.99-1.3	102 (81-126)	1.07 (0.86-1.3	-0.15 (-0.58-0.28

		6)		2)	)
Portugal	87 (73-102)	0.62 (0.52-0.73)	99 (81-121)	0.45 (0.37-0.53)	-1.53 (-1.76--1.31)
Democratic Republic of the Congo	50 (17-96)	0.32 (0.11-0.62)	86 (33-170)	0.22 (0.09-0.43)	-1.44 (-2.04--0.83)
Bosnia and Herzegovina	38 (26-58)	0.94 (0.65-1.42)	85 (58-120)	1.37 (0.92-1.92)	1.45 (1.17-1.73)
Nepal	22 (12-41)	0.28 (0.15-0.51)	85 (55-139)	0.41 (0.26-0.67)	1.27 (1.11-1.43)
Slovakia	58 (45-74)	0.97 (0.76-1.23)	82 (59-113)	0.88 (0.63-1.20)	-0.26 (-0.35--0.16)
Uruguay	87 (73-100)	2.24 (1.90-2.56)	82 (67-98)	1.56 (1.30-1.84)	-1.62 (-1.90--1.33)
Ireland	132 (112-153)	3.12 (2.64-3.60)	75 (56-95)	0.91 (0.69-1.14)	-4.50 (-4.92--4.08)
Ghana	24 (9-44)	0.42 (0.16-0.79)	72 (29-136)	0.46 (0.18-0.85)	0.29 (0.08-0.51)
Angola	22 (9-39)	0.58 (0.24-1.02)	71 (37-115)	0.60 (0.31-0.96)	0.05 (-0.18-0.28)
Zimbabwe	35 (25-46)	0.99 (0.71-1.31)	69 (47-97)	1.10 (0.75-1.55)	0.20 (-0.05-0.45)
Democratic People's Republic of Korea	34 (23-52)	0.19 (0.13-0.29)	67 (47-98)	0.20 (0.14-0.28)	0.13 (0.07-0.18)
Paraguay	21 (16-27)	0.98 (0.74-1.26)	67 (47-90)	1.17 (0.82-1.56)	0.58 (0.47-0.68)
Lebanon	30 (15-57)	1.38 (0.66-2.64)	66 (51-85)	1.07 (0.84-1.37)	-0.74 (-0.98--0.50)
Kenya	23 (11-36)	0.29 (0.14-0.47)	63 (34-93)	0.28 (0.15-0.41)	-0.72 (-0.91--0.53)
Lithuania	38 (32-44)	0.84	63 (51-76)	1.21	0.95

		(0.71-0.98)		(0.98-1.43)	(0.68-1.23)
Uzbekistan	5 (4-7)	0.04 (0.03-0.06)	62 (47-79)	0.23 (0.18-0.30)	6.28 (5.60-6.95)
Azerbaijan	13 (10-18)	0.25 (0.19-0.35)	56 (29-102)	0.54 (0.29-0.92)	3.11 (2.71-3.51)
Dominican Republic	18 (14-24)	0.56 (0.41-0.72)	55 (38-77)	0.56 (0.39-0.78)	-0.08 (-0.35-0.20)
Mozambique	19 (5-41)	0.34 (0.09-0.74)	53 (17-110)	0.46 (0.15-0.94)	1.22 (1.12-1.32)
Peru	19 (14-27)	0.16 (0.12-0.23)	50 (35-73)	0.15 (0.10-0.22)	-0.59 (-0.83--0.35)
Algeria	7 (5-10)	0.07 (0.04-0.09)	48 (33-68)	0.15 (0.10-0.20)	3.21 (2.93-3.50)
C 么 te d'Ivoire	18 (6-35)	0.46 (0.14-0.88)	48 (16-86)	0.41 (0.14-0.75)	-1.23 (-1.59--0.86)
Israel	44 (36-52)	0.90 (0.75-1.07)	47 (38-58)	0.38 (0.30-0.46)	-3.16 (-3.38--2.94)
Zambia	13 (7-21)	0.50 (0.28-0.83)	44 (16-86)	0.64 (0.25-1.19)	0.79 (0.33-1.25)
Ecuador	21 (17-25)	0.40 (0.33-0.48)	43 (30-57)	0.26 (0.18-0.35)	-1.00 (-1.36--0.64)
Latvia	32 (27-37)	0.89 (0.76-1.03)	42 (34-50)	1.18 (0.96-1.39)	0.26 (-0.09-0.62)
Ethiopia	22 (7-50)	0.11 (0.03-0.23)	41 (15-76)	0.09 (0.03-0.17)	-0.47 (-0.92--0.02)
Iraq	12 (8-17)	0.14 (0.09-0.21)	41 (29-57)	0.17 (0.12-0.22)	0.31 (0.22-0.40)
Cameroon	13 (5-25)	0.30 (0.12-0.55)	40 (18-66)	0.30 (0.14-0.49)	-0.36 (-0.59--0.12)

Costa Rica	14 (11-17)	0.82 (0.66-0.99)	40 (31-50)	0.72 (0.57-0.91)	-0.94 (-1.24--0.64)
Madagascar	32 (13-61)	0.65 (0.25-1.23)	39 (16-70)	0.32 (0.13-0.55)	-2.56 (-2.99--2.12)
Malawi	13 (4-26)	0.38 (0.11-0.76)	38 (15-72)	0.52 (0.20-0.98)	0.80 (0.65-0.95)
Montenegro	20 (15-27)	3.21 (2.45-4.28)	36 (25-49)	3.71 (2.55-4.99)	0.68 (0.60-0.75)
Morocco	6 (3-9)	0.04 (0.02-0.06)	36 (22-53)	0.10 (0.06-0.15)	3.48 (3.27-3.68)
Singapore	18 (15-21)	0.81 (0.67-0.97)	36 (29-45)	0.42 (0.33-0.52)	-2.09 (-2.24--1.93)
Jordan	8 (5-10)	0.55 (0.40-0.75)	35 (25-47)	0.45 (0.32-0.60)	-0.73 (-0.90--0.56)
North Macedonia	16 (12-20)	0.88 (0.65-1.15)	35 (21-55)	1.06 (0.65-1.61)	0.28 (0.02-0.54)
Republic of Moldova	14 (12-17)	0.32 (0.27-0.38)	35 (28-42)	0.59 (0.47-0.71)	1.92 (1.61-2.23)
Uganda	15 (5-32)	0.25 (0.08-0.52)	35 (14-63)	0.23 (0.10-0.42)	-0.95 (-1.22--0.68)
Rwanda	22 (12-37)	0.87 (0.48-1.47)	33 (17-59)	0.60 (0.32-1.06)	-1.87 (-2.26--1.49)
Cyprus	21 (15-30)	3.01 (2.10-4.24)	32 (22-43)	1.51 (1.03-1.98)	-2.65 (-2.88--2.43)
Estonia	23 (19-26)	1.10 (0.93-1.29)	31 (25-38)	1.23 (0.99-1.48)	-0.24 (-0.60-0.13)
Slovenia	26 (22-31)	1.07 (0.90-1.24)	31 (23-40)	0.71 (0.55-0.90)	-1.54 (-1.93--1.14)
Cambodia	8 (4-13)	0.20 (0.11-0.3	30 (18-52)	0.28 (0.16-0.5	1.17 (1.13-1.21)

		4)		0)	
Bolivia (Plurinational State of)	9 (6-16)	0.29 (0.18-0.49)	25 (17-37)	0.28 (0.19-0.41)	-0.03 (-0.16-0.10)
Turkmenistan	7 (6-9)	0.35 (0.28-0.44)	25 (17-38)	0.58 (0.40-0.89)	1.52 (1.31-1.73)
Tunisia	4 (2-5)	0.07 (0.04-0.10)	24 (15-38)	0.19 (0.11-0.29)	3.43 (3.12-3.73)
Senegal	11 (3-22)	0.33 (0.09-0.65)	23 (8-44)	0.28 (0.10-0.54)	-0.89 (-1.07--0.71)
Albania	8 (6-10)	0.41 (0.31-0.53)	22 (14-33)	0.51 (0.31-0.76)	0.90 (0.76-1.04)
Haiti	12 (7-23)	0.39 (0.23-0.74)	22 (13-40)	0.33 (0.19-0.62)	-0.56 (-0.65--0.47)
Papua New Guinea	7 (4-12)	0.38 (0.22-0.62)	21 (13-34)	0.37 (0.21-0.58)	-0.28 (-0.39--0.16)
Sudan	3 (1-8)	0.03 (0.01-0.08)	21 (12-34)	0.10 (0.06-0.16)	4.13 (3.87-4.40)
Syrian Arab Republic	8 (5-12)	0.15 (0.10-0.22)	21 (14-29)	0.16 (0.10-0.22)	0.05 (-0.05-0.15)
Sri Lanka	10 (8-14)	0.11 (0.08-0.15)	20 (12-31)	0.08 (0.05-0.12)	-0.87 (-1.01--0.73)
Yemen	2 (1-4)	0.04 (0.02-0.08)	20 (11-34)	0.13 (0.07-0.22)	4.17 (3.80-4.55)
Burkina Faso	7 (2-17)	0.17 (0.04-0.38)	19 (5-36)	0.20 (0.05-0.38)	0.58 (0.37-0.79)
Honduras	4 (3-7)	0.23 (0.15-0.35)	19 (12-29)	0.31 (0.20-0.47)	1.22 (1.11-1.33)
Trinidad and Tobago	13 (11-15)	1.55 (1.26-1.89)	19 (13-27)	0.98 (0.69-1.36)	-2.21 (-2.60--1.81)
Congo	8 (4-12)	0.72	18 (9-29)	0.67	-0.42

		(0.35-1.17)		(0.36-1.09)	(-0.79--0.05)
Panama	10 (8-11)	0.66 (0.54-0.80)	18 (12-24)	0.40 (0.28-0.54)	-2.23 (-2.50--1.97)
Puerto Rico	24 (18-29)	0.66 (0.51-0.82)	18 (13-24)	0.25 (0.19-0.33)	-3.90 (-4.18--3.63)
Togo	7 (2-13)	0.57 (0.19-1.10)	18 (6-33)	0.46 (0.17-0.86)	-1.13 (-1.29--0.96)
Guinea	8 (2-16)	0.24 (0.06-0.49)	17 (5-33)	0.30 (0.08-0.61)	0.59 (0.47-0.70)
Kyrgyzstan	3 (2-3)	0.09 (0.07-0.11)	17 (13-21)	0.33 (0.26-0.42)	5.30 (4.61-5.98)
United Arab Emirates	2 (1-4)	0.35 (0.20-0.56)	16 (12-22)	0.34 (0.24-0.46)	1.23 (0.57-1.89)
Mali	4 (1-10)	0.11 (0.03-0.27)	15 (4-32)	0.18 (0.04-0.40)	1.83 (1.57-2.09)
Jamaica	9 (7-11)	0.47 (0.37-0.58)	13 (9-19)	0.43 (0.30-0.61)	-0.52 (-0.95--0.09)
Saudi Arabia	1 (1-1)	0.01 (0.01-0.02)	13 (8-18)	0.04 (0.03-0.06)	4.63 (4.08-5.18)
Chad	5 (2-11)	0.19 (0.05-0.41)	12 (3-23)	0.21 (0.05-0.42)	-0.00 (-0.18-0.17)
Lao People's Democratic Republic	5 (3-8)	0.26 (0.14-0.40)	12 (8-18)	0.31 (0.21-0.46)	0.71 (0.65-0.77)
Kuwait	3 (3-4)	0.40 (0.33-0.47)	11 (8-14)	0.34 (0.25-0.44)	0.15 (-1.05-1.36)
Sierra Leone	8 (2-16)	0.37 (0.10-0.77)	11 (4-23)	0.28 (0.09-0.57)	-1.07 (-1.21--0.92)
Guatemala	5 (4-6)	0.14 (0.11-0.18)	10 (7-13)	0.09 (0.07-0.11)	-2.17 (-2.44--1.88)

South Sudan	10 (2-20)	0.40 (0.10-0.79)	10 (2-20)	0.25 (0.07-0.52)	-1.87 (-2.17--1.56)
Benin	4 (1-9)	0.22 (0.07-0.46)	9 (3-18)	0.17 (0.05-0.34)	-1.15 (-1.30--1.01)
Burundi	11 (4-19)	0.45 (0.16-0.83)	9 (3-19)	0.18 (0.06-0.38)	-3.40 (-4.03--2.76)
Afghanistan	2 (1-3)	0.02 (0.01-0.04)	8 (4-15)	0.08 (0.03-0.13)	4.88 (4.52-5.25)
Botswana	4 (3-7)	0.85 (0.52-1.49)	8 (5-11)	0.60 (0.34-0.83)	-1.49 (-1.70--1.28)
Luxembourg	10 (8-12)	1.78 (1.46-2.12)	8 (6-10)	0.77 (0.60-0.96)	-3.36 (-3.64--3.08)
Palestine	2 (1-3)	0.26 (0.16-0.37)	8 (6-10)	0.31 (0.22-0.42)	0.47 (0.40-0.55)
Somalia	5 (1-12)	0.21 (0.05-0.46)	8 (2-19)	0.12 (0.03-0.29)	-2.06 (-2.30--1.83)
Central African Republic	5 (2-11)	0.46 (0.16-0.93)	7 (3-15)	0.31 (0.12-0.63)	-1.56 (-1.78--1.35)
El Salvador	4 (3-5)	0.12 (0.08-0.15)	7 (5-10)	0.12 (0.09-0.17)	0.02 (-0.15-0.20)
Fiji	4 (3-5)	1.00 (0.76-1.30)	7 (5-10)	0.90 (0.63-1.24)	-0.62 (-0.75--0.50)
Gabon	4 (2-6)	0.62 (0.32-0.98)	7 (4-10)	0.60 (0.34-0.97)	-0.32 (-0.45--0.20)
Libya	1 (1-2)	0.05 (0.03-0.07)	7 (4-12)	0.12 (0.07-0.20)	3.97 (3.58-4.36)
Namibia	4 (3-7)	0.83 (0.49-1.30)	7 (5-11)	0.63 (0.42-0.93)	-1.29 (-1.53--1.05)
Eritrea	3 (1-8)	0.23 (0.08-0.6	6 (2-14)	0.19 (0.07-0.4	-1.18 (-1.43--0.9

		0)		1)	3)
Lesotho	3 (2-6)	0.41 (0.20-0.76)	6 (2-11)	0.57 (0.27-1.07)	1.41 (1.25-1.57)
Mauritania	4 (1-6)	0.34 (0.13-0.62)	6 (2-12)	0.27 (0.09-0.57)	-1.24 (-1.51--0.98)
Mongolia	1 (1-2)	0.10 (0.06-0.15)	6 (4-8)	0.23 (0.16-0.32)	3.28 (2.97-3.59)
Niger	2 (0-6)	0.09 (0.02-0.22)	6 (1-16)	0.09 (0.01-0.21)	-0.33 (-0.46--0.20)
Brunei Darussalam	2 (1-3)	2.12 (1.45-2.87)	5 (4-6)	1.35 (1.00-1.75)	-1.10 (-1.29--0.90)
Guyana	2 (1-2)	0.44 (0.35-0.55)	5 (3-7)	0.75 (0.52-1.05)	1.20 (0.46-1.94)
Iceland	5 (5-7)	1.86 (1.55-2.22)	5 (4-6)	0.81 (0.63-1.04)	-3.18 (-3.51--2.86)
Liberia	3 (1-6)	0.28 (0.07-0.55)	5 (1-11)	0.23 (0.06-0.48)	-0.85 (-1.01--0.68)
Mauritius	3 (3-4)	0.44 (0.37-0.52)	5 (4-6)	0.30 (0.25-0.36)	-1.19 (-1.76--0.60)
Tajikistan	3 (2-4)	0.12 (0.08-0.16)	5 (3-7)	0.08 (0.05-0.12)	-1.34 (-1.60--1.09)
Gambia	2 (0-3)	0.46 (0.12-0.95)	4 (1-8)	0.41 (0.15-0.76)	-0.84 (-1.05--0.62)
Malta	5 (4-5)	1.07 (0.89-1.26)	4 (3-5)	0.43 (0.34-0.54)	-3.54 (-3.91--3.16)
Nicaragua	1 (1-2)	0.10 (0.07-0.13)	4 (3-6)	0.09 (0.07-0.12)	-0.10 (-0.26-0.05)
Oman	0 (0-1)	0.05 (0.03-0.09)	4 (2-7)	0.15 (0.08-0.26)	4.02 (3.61-4.44)
Suriname	2 (2-3)	0.87	4 (3-6)	0.61	-1.40

		(0.65-1.13)		(0.40-0.90)	(-1.66--1.15)
Djibouti	1 (0-1)	0.62 (0.28-1.10)	3 (1-5)	0.51 (0.20-0.87)	-0.91 (-1.07--0.75)
Equatorial Guinea	1 (0-2)	0.46 (0.18-0.88)	3 (1-6)	0.58 (0.24-1.09)	0.73 (0.56-0.90)
Qatar	1 (0-1)	0.39 (0.27-0.55)	3 (2-6)	0.26 (0.14-0.46)	-1.51 (-1.92--1.09)
Andorra	2 (1-2)	2.74 (1.60-4.19)	2 (1-3)	1.33 (0.77-2.16)	-2.29 (-2.54--2.04)
Bahamas	1 (1-2)	0.90 (0.70-1.12)	2 (2-3)	0.62 (0.45-0.81)	-1.35 (-1.67--1.02)
Bahrain	0 (0-1)	0.23 (0.17-0.32)	2 (1-3)	0.23 (0.16-0.32)	-0.03 (-0.40-0.34)
Barbados	2 (2-3)	0.68 (0.52-0.84)	2 (1-3)	0.41 (0.29-0.56)	-2.62 (-3.09--2.15)
Bermuda	2 (2-2)	3.10 (2.44-3.82)	2 (1-2)	1.11 (0.83-1.49)	-3.37 (-3.54--3.20)
Bhutan	0 (0-1)	0.16 (0.07-0.25)	2 (1-3)	0.31 (0.14-0.54)	2.37 (2.31-2.43)
Comoros	1 (0-2)	0.54 (0.14-1.11)	2 (1-4)	0.38 (0.11-0.76)	-1.61 (-1.90--1.32)
Eswatini	1 (1-2)	0.49 (0.28-0.79)	2 (1-3)	0.36 (0.21-0.55)	-1.01 (-1.27--0.74)
Guinea-Bissau	1 (0-2)	0.24 (0.08-0.49)	2 (1-4)	0.26 (0.09-0.49)	0.19 (0.01-0.38)
Monaco	1 (1-2)	2.07 (1.50-2.74)	2 (1-2)	1.61 (1.11-2.31)	-0.83 (-0.99--0.67)
Saint Lucia	2 (1-2)	1.98 (1.54-2.48)	2 (1-3)	0.84 (0.62-1.13)	-3.57 (-3.89--3.24)

Solomon Islands	1 (0-1)	0.52 (0.27-0.94)	2 (1-3)	0.55 (0.32-0.89)	0.14 (-0.05-0.32)
Timor-Leste	0 (0-1)	0.16 (0.08-0.25)	2 (1-3)	0.21 (0.13-0.36)	1.03 (0.92-1.13)
Belize	0 (0-0)	0.23 (0.18-0.28)	1 (0-1)	0.18 (0.14-0.24)	-1.43 (-2.14--0.71)
Cabo Verde	1 (0-1)	0.28 (0.08-0.58)	1 (1-2)	0.30 (0.13-0.55)	-0.36 (-0.71--0.00)
Dominica	0 (0-1)	0.71 (0.48-1.05)	1 (0-1)	0.69 (0.49-0.96)	-0.38 (-0.50--0.26)
Grenada	1 (1-1)	1.14 (0.89-1.46)	1 (1-1)	0.85 (0.64-1.11)	-1.99 (-2.77--1.20)
Guam	1 (1-2)	1.74 (1.33-2.23)	1 (1-2)	0.63 (0.50-0.79)	-3.32 (-3.47--3.17)
Maldives	0 (0-0)	0.29 (0.15-0.45)	1 (0-1)	0.22 (0.08-0.39)	-1.45 (-1.62--1.28)
Micronesia (Federated States of)	1 (0-1)	1.11 (0.72-1.68)	1 (0-1)	0.98 (0.65-1.37)	-0.62 (-0.69--0.54)
Saint Vincent and the Grenadines	0 (0-1)	0.55 (0.43-0.70)	1 (0-1)	0.44 (0.34-0.56)	-1.30 (-1.59--1.01)
Samoa	1 (1-1)	1.02 (0.67-1.50)	1 (1-2)	0.84 (0.59-1.13)	-0.86 (-0.97--0.75)
Tonga	1 (0-1)	1.06 (0.80-1.43)	1 (1-1)	1.09 (0.71-1.50)	-0.12 (-0.31-0.08)
United States Virgin Islands	1 (0-1)	0.74 (0.52-1.02)	1 (1-1)	0.43 (0.29-0.61)	-2.01 (-2.24--1.77)
Vanuatu	0 (0-1)	0.53 (0.29-0.90)	1 (1-1)	0.45 (0.28-0.70)	-0.88 (-1.03--0.73)
American Samoa	0 (0-0)	1.01 (0.75-1.2	0 (0-0)	0.71 (0.55-0.9	-1.47 (-1.67--1.2

		7)		3)	7)
Antigua and Barbuda	0 (0-1)	0.72 (0.56-0.91)	0 (0-1)	0.43 (0.32-0.58)	-2.40 (-2.73--2.07)
Cook Islands	0 (0-0)	1.00 (0.73-1.31)	0 (0-0)	0.78 (0.41-1.38)	-0.97 (-1.06--0.88)
Greenland	1 (0-1)	1.75 (1.42-2.15)	0 (0-1)	0.73 (0.56-0.96)	-2.97 (-3.14--2.80)
Kiribati	0 (0-0)	0.18 (0.14-0.22)	0 (0-0)	0.20 (0.14-0.27)	0.20 (0.01-0.40)
Marshall Islands	0 (0-0)	0.68 (0.41-1.13)	0 (0-0)	0.70 (0.41-1.10)	-0.01 (-0.05-0.04)
Nauru	0 (0-0)	1.70 (1.24-2.32)	0 (0-0)	1.45 (0.99-2.03)	-0.69 (-0.76--0.61)
Niue	0 (0-0)	0.87 (0.62-1.24)	0 (0-0)	0.79 (0.55-1.07)	-0.61 (-0.72--0.49)
Northern Mariana Islands	0 (0-0)	1.38 (1.04-1.82)	0 (0-1)	0.79 (0.58-1.05)	-2.78 (-3.12--2.43)
Palau	0 (0-0)	0.84 (0.60-1.14)	0 (0-0)	0.71 (0.51-0.97)	-0.72 (-0.79--0.65)
Saint Kitts and Nevis	0 (0-0)	0.77 (0.58-0.99)	0 (0-0)	0.42 (0.30-0.60)	-2.46 (-2.71--2.21)
San Marino	0 (0-1)	1.33 (1.00-1.73)	0 (0-1)	0.62 (0.37-1.00)	-1.49 (-1.87--1.12)
Sao Tome and Principe	0 (0-0)	0.17 (0.05-0.36)	0 (0-1)	0.25 (0.10-0.51)	1.09 (0.91-1.26)
Seychelles	0 (0-0)	0.39 (0.29-0.49)	0 (0-1)	0.35 (0.21-0.54)	-0.33 (-0.51--0.15)
Tokelau	0 (0-0)	0.90 (0.64-1.28)	0 (0-0)	0.77 (0.53-1.09)	-0.77 (-0.92--0.62)
Tuvalu	0 (0-0)	0.80	0 (0-0)	0.80	-0.21

		(0.52-1.23)		(0.56-1.10)	(-0.30--0.11)

Notes: ASMR, Age-standardized mortality rate; EAPC, Estimated annual percentage change.

© 2026 Jin X. et al.
